# Supplementary material for: β-arrestin 2 as an activator of cGAS-STING signaling and target of viral immune evasion
Source: Nat Commun. 2020 Nov 26;11:6000. doi: 10.1038/s41467-020-19849-9 (PMC7691508; doi:10.1038/s41467-020-19849-9)
Supplement: Supplementary file 1 — Supplementary Information [file 41467_2020_19849_MOESM1_ESM.pdf]

Supplementary Information for

β-arrestin 2 as an activator of cGAS-STING signaling and target of viral immune evasion

Yihua Zhang[1,†], Manman Li[1,†], Liuyan Li[1], Gui Qian[1], Yu Wang[2], Zijuan Chen[1], Jing Liu[1], Chao Fang[3], Feng Huang[4], Daqiao Guo[3], Quanming Zou[2], Yiwei Chu[1] & Dapeng Yan[1]*

[1]Department of Immunology, School of Basic Medical Sciences & Shanghai Public Health Clinical Center, Key Laboratory of Medical Molecular Virology of MOE/MOH, Fudan University, Shanghai 200032, China
[2]Department of Microbiology and Biochemical Pharmacy, National Engineering Research Centre of Immunological Products, College of Pharmacy, Army Medical University, Chongqing 400038, China
[3]Department of Vascular Surgery, Zhongshan Hospital Affiliated to Fudan University, Institute of Vascular Surgery, Fudan University, Shanghai 200032, China
[4]Beijing Hospital of Traditional Chinese Medicine, Capital Medical University, Beijing 100010, China
†These authors contributed equally to this work.
* Correspondence should be addressed to D. Y. (dapengyan@fudan.edu.cn)

This PDF file includes:

Supplementary Fig. 1 to 6
Supplementary Table 1

Supplementary Figures

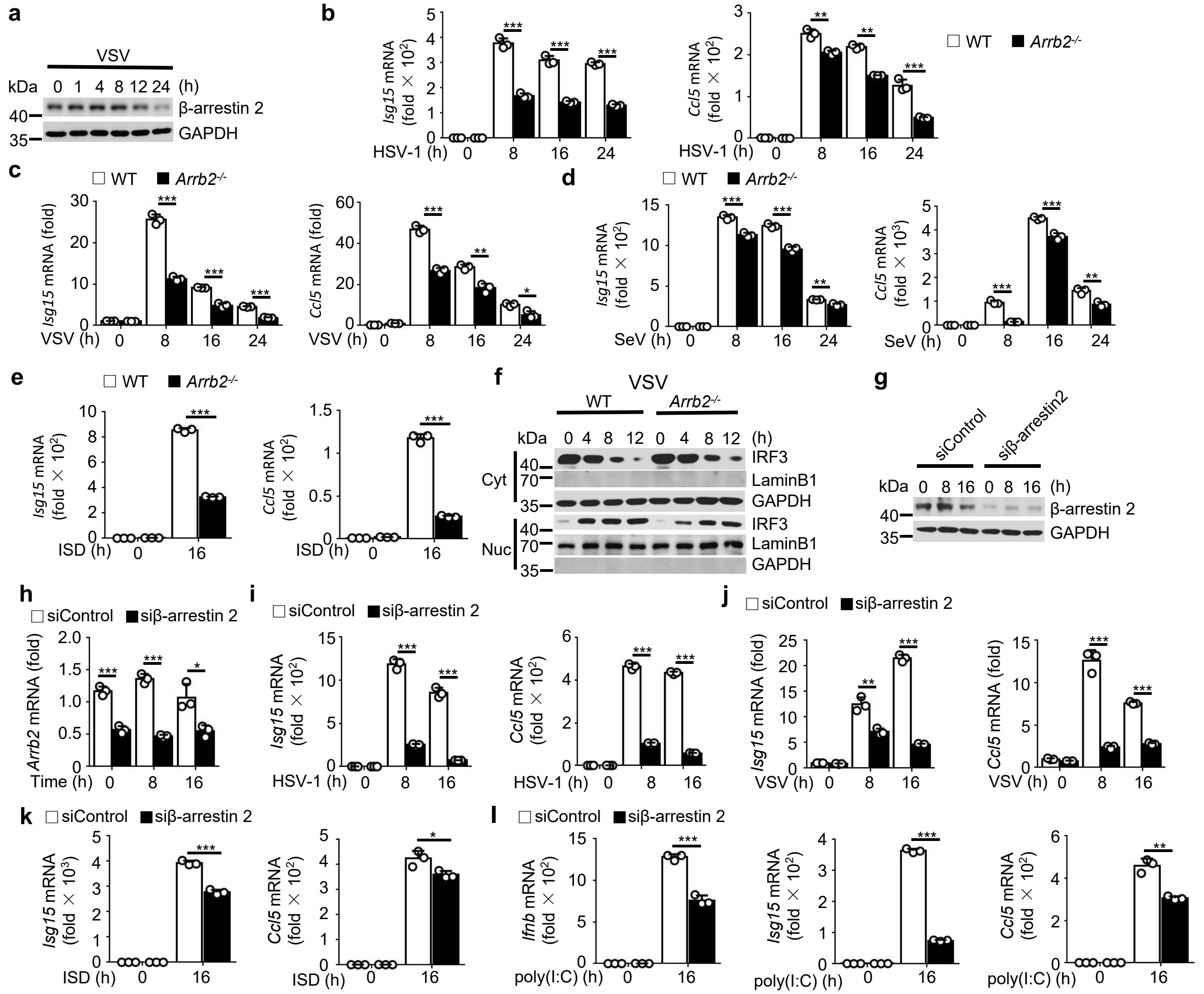

Supplementary Fig. 1: β-arrestin 2 promoted the antiviral immune response. (**a**) Immunoblot of lysates of peritoneal macrophages infected with VSV for indicated times. (**b–e**) *Isg15* and *Cxcl10* mRNA levels in WT or *Arrb2*$^{-/-}$ mouse peritoneal macrophages stimulated with HSV-1 (**b**) (****P* < 0.0001, < 0.0001, < 0.0001 in sequence, left panel; ***P* = 0.0025, ****P* < 0.0001, ***P* = 0.0007, right panel), VSV (**c**) (****P* < 0.0001, = 0.0001, < 0.0001 in sequence, left panel; ****P* < 0.0001, ***P* = 0.0020, **P* = 0.0125, right panel), SeV (**d**) (****P* = 0.000, 0.0002 in sequence, ***P* = 0.0024, left panel; ****P* < 0.0001, =0.0009 in sequence, ***P* = 0.0035, right panel), or ISD (**e**) (****P* < 0.0001 in all panels) for indicated times. (**f**) Immunoblot analysis of nuclear and cytoplasmic fractions as in WT or *Arrb2*$^{-/-}$ mouse peritoneal macrophages infected with VSV for indicated times. (**g**) Immunoblot of lysates of RAW264.7 cells treated with control siRNA or siβ-arrestin 2 and infected with HSV-1 for indicated times. (**h**) *Arrb2*$^{-/-}$ mRNA levels in RAW264.7 cells treated with control siRNA or siβ-arrestin 2 and infected with HSV-1 for indicated times (****P* = 0.0003, ****P* < 0.0001, **P* = 0.0164). (**i–l**) *Ifnb*, *Isg15*, and *Cxcl10* mRNA levels in RAW264.7 cells transfected with control siRNA or β-arrestin 2 siRNA and then stimulated with HSV-1 (**i**) (****P* < 0.0001 in all panels), VSV (**j**) (***P* = 0.0035, ****P* < 0.0001 left panel; ****P* = 0.0001, ****P* < 0.0001, right panel), ISD (**k**) (****P* = 0.0001, left panel; ***P* = 0.0306, right panel), or poly(I:C) (**l**) (****P* = 0.0002, left panel; ****P* < 0.0001, middle panel; ***P* = 0.0014, right panel) for indicated times. Data are representative of at least three independent experiments (mean ± SEM in **b–e, h–l,** *n* = 3). Two-tailed unpaired Student's *t*-test.

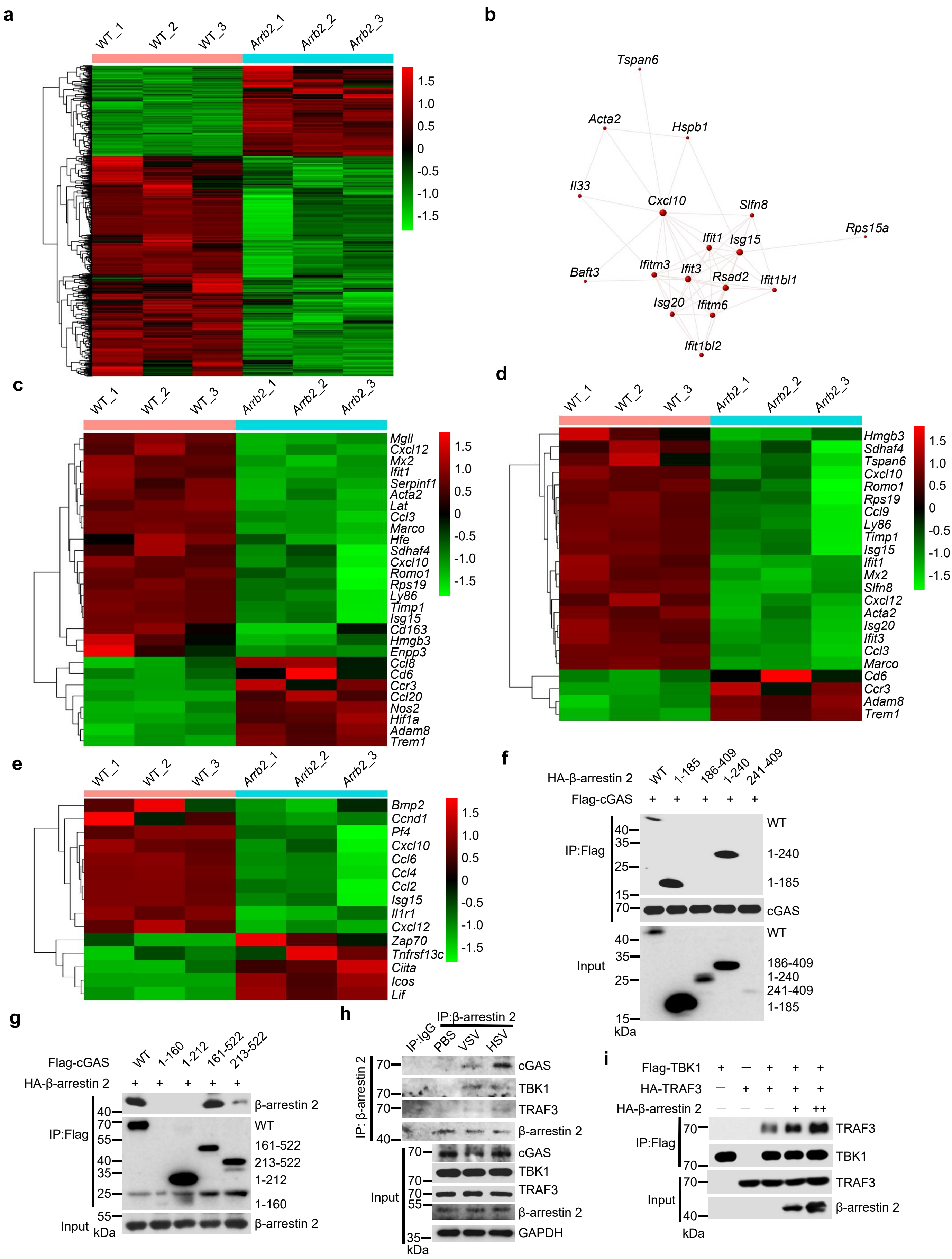

a
WT_1 WT_2 WT_3 Arrb2_1 Arrb2_2 Arrb2_3
1.5 1.0 0.5 0 -0.5 -1.0 -1.5
b
Tspan6 Acta2 Hspb1 Il33 Cxcl10 Slfn8 Rps15a Ifit1 Isg15 Baft3 Ifitm3 Ifit3 Rsad2 Ifit1bl1 Isg20 Ifitm6 Ifit1bl2
c
WT_1 WT_2 WT_3 Arrb2_1 Arrb2_2 Arrb2_3
Mgll Cxcl12 Mx2 Ifit1 Serpinf1 Acta2 Lat Ccl3 Marco Hfe Sdhaf4 Cxcl10 Romo1 Rps19 Ly86 Timp1 Isg15 Cd163 Hmgb3 Enpp3 Ccl8 Cd6 Ccr3 Ccl20 Nos2 Hif1a Adam8 Trem1
1.5 1.0 0.5 0 -0.5 -1.0 -1.5
d
WT_1 WT_2 WT_3 Arrb2_1 Arrb2_2 Arrb2_3
Hmgb3 Sdhaf4 Tspan6 Cxcl10 Romo1 Rps19 Ccl9 Ly86 Timp1 Isg15 Ifit1 Mx2 Slfn8 Cxcl12 Acta2 Isg20 Ifit3 Ccl3 Marco Cd6 Ccr3 Adam8 Trem1
1.5 1.0 0.5 0 -0.5 -1.0 -1.5
e
WT_1 WT_2 WT_3 Arrb2_1 Arrb2_2 Arrb2_3
Bmp2 Ccnd1 Pf4 Cxcl10 Ccl6 Ccl4 Ccl2 Isg15 Il1r1 Cxcl12 Zap70 Tnfrsf13c Ciita Icos Lif
1.5 1.0 0.5 0 -0.5 -1.0 -1.5
f
HA-β-arrestin 2 WT 1-185 186-409 1-240 241-409
Flag-cGAS + + + + +
IP:Flag
40 35 25 15 70
WT 1-240 1-185 cGAS
Input
40 35 25 15
WT 186-409 1-240 241-409 1-185
kDa
g
Flag-cGAS WT 1-160 1-212 161-522 213-522
HA-β-arrestin 2 + + + + +
IP:Flag
40 70 55 40 35 25
β-arrestin 2 WT 161-522 213-522 1-212 1-160
Input 55
β-arrestin 2
kDa
h
IP:IgG IP:β-arrestin 2 PBS VSV HSV
IP: β-arrestin 2
70 70 70 40
cGAS TBK1 TRAF3 β-arrestin 2
Input
70 70 70 55 35
cGAS TBK1 TRAF3 β-arrestin 2 GAPDH
kDa
i
Flag-TBK1 + — + + +
HA-TRAF3 — + + + +
HA-β-arrestin 2 — — — + ++
IP:Flag
70 70
TRAF3 TBK1
Input
70 40
TRAF3 β-arrestin 2
kDa

Supplementary Fig. 2: β-arrestin 2 mediated the modulation of host antiviral transcriptional program during HSV-1 infection. (**a**) Heatmap shown the differentially expressed genes (fold change > 2 and p-value < 0.05). (**b**) Network model based on differential expressed genes. (**c**) Heatmap of the differential expressed genes involved in antiviral signaling pathway. (**d**) Heatmap of differential genes involved in the terms in red as in Figure 4**b**. (**e**) Heatmap of differential genes involved in the terms in red as in Figure 4**c**. (**f**) Co-immunoassay of lysates of HEK293T cells expressing β-arrestin 2 trunctions and cGAS. (**g**) Co-immunoassay of HEK293T cells expressing cGAS trunctions and β-arrestin 2. (**h**) Immunoassay of lysates of peritoneal macrophages infected with HSV-1 or VSV. (**i**) Immunoassay of HEK293T cells expressing various vectors. Data are representative of at least three independent experiments.

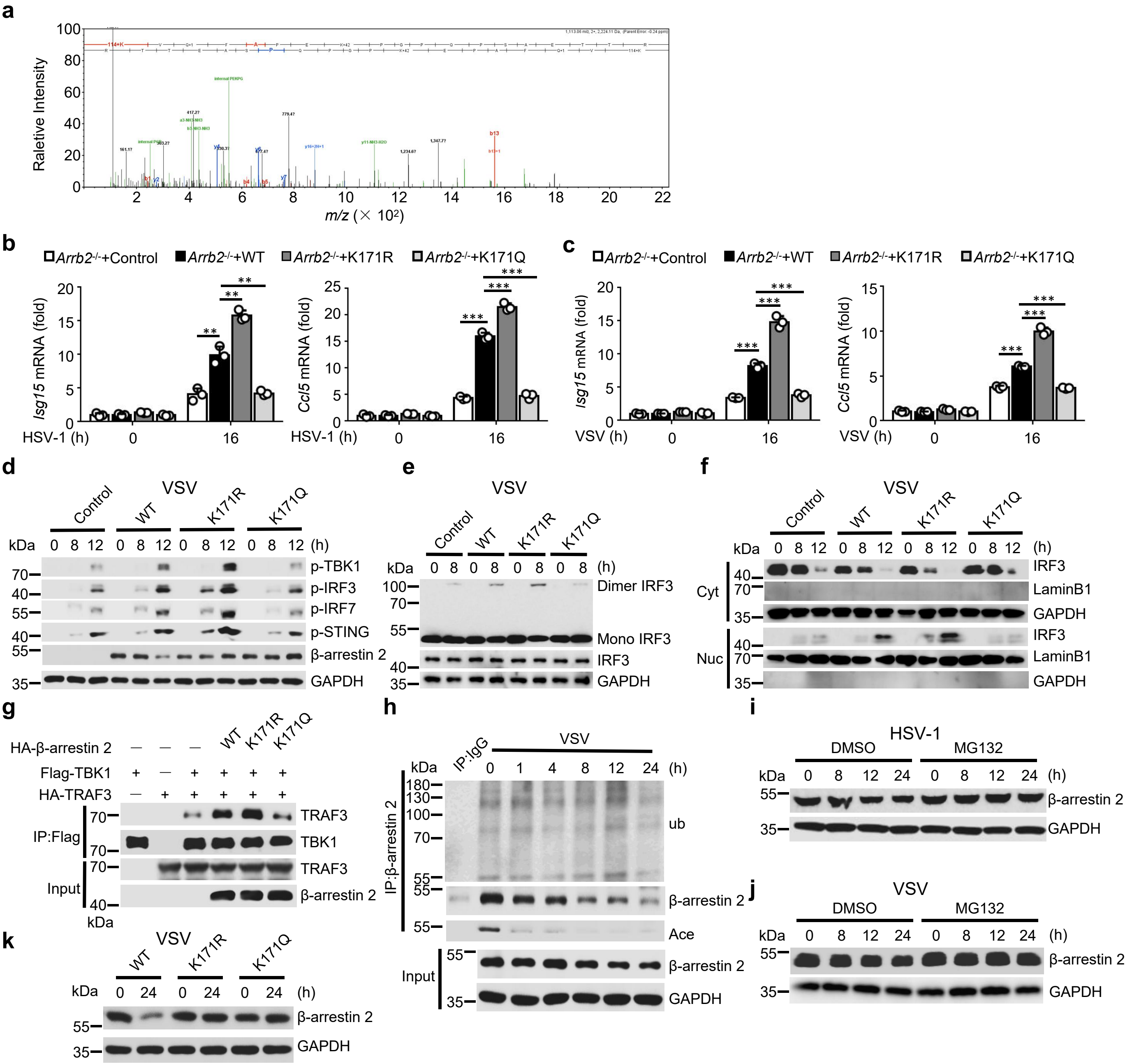

Supplementary Fig. 3: Lys171 was the pivotal regulation residue of β-arrestin 2. (**a**) Mass spectrometry analysis of a β-arrestin 2 peptide modified with acetylation. (**b, c**) *Ifnb*, *Isg15*, and *Il6* mRNA levels in *Arrb2*$^{-/-}$ MEFs transfected with control vector, β-arrestin 2, or β-arrestin 2 mutants and infected with HSV-1 (**b**) (***P* = 0.0030, 0.0025, 0.0018 in sequence, left panel; ****P* < 0.0001, = 0.0006, < 0.0001 in sequence, right panel) or VSV (**c**) (****P* < 0.0001, = 0.0003, < 0.0001 in sequence, left panel; ****P* < 0.0001, < 0.0001, < 0.0001 in sequence, right panel) for 16 h. (**d**) Immunoassay of lysates of *Arrb2*$^{-/-}$ MEFs transfected as in (**c**) and infected with VSV for indicated times. (**e**) Immunoblot analysis of monomeric and dimeric IRF3 as in (**d**). (**f**) Immunoblot analysis of nuclear and cytoplasmic fractions as in (**d**). (**g**) Immunoassay of HEK293T cells expressing various vectors. (**h**) Immunoassay of lysates of peritoneal macrophages infected with VSV for indicated times. (**i,j**) Immunoassay of lysates of peritoneal macrophages infected with HSV-1 (**i**) and VSV (**j**) for indicated times and treated with MG132 before lysed. (**k**)Immunoassay of lysates as in (**d**) for 24 h. Data are representative of at least three independent experiments (mean ± SEM in **b, c,** *n* = 3). Two-tailed unpaired Student's *t*-test.

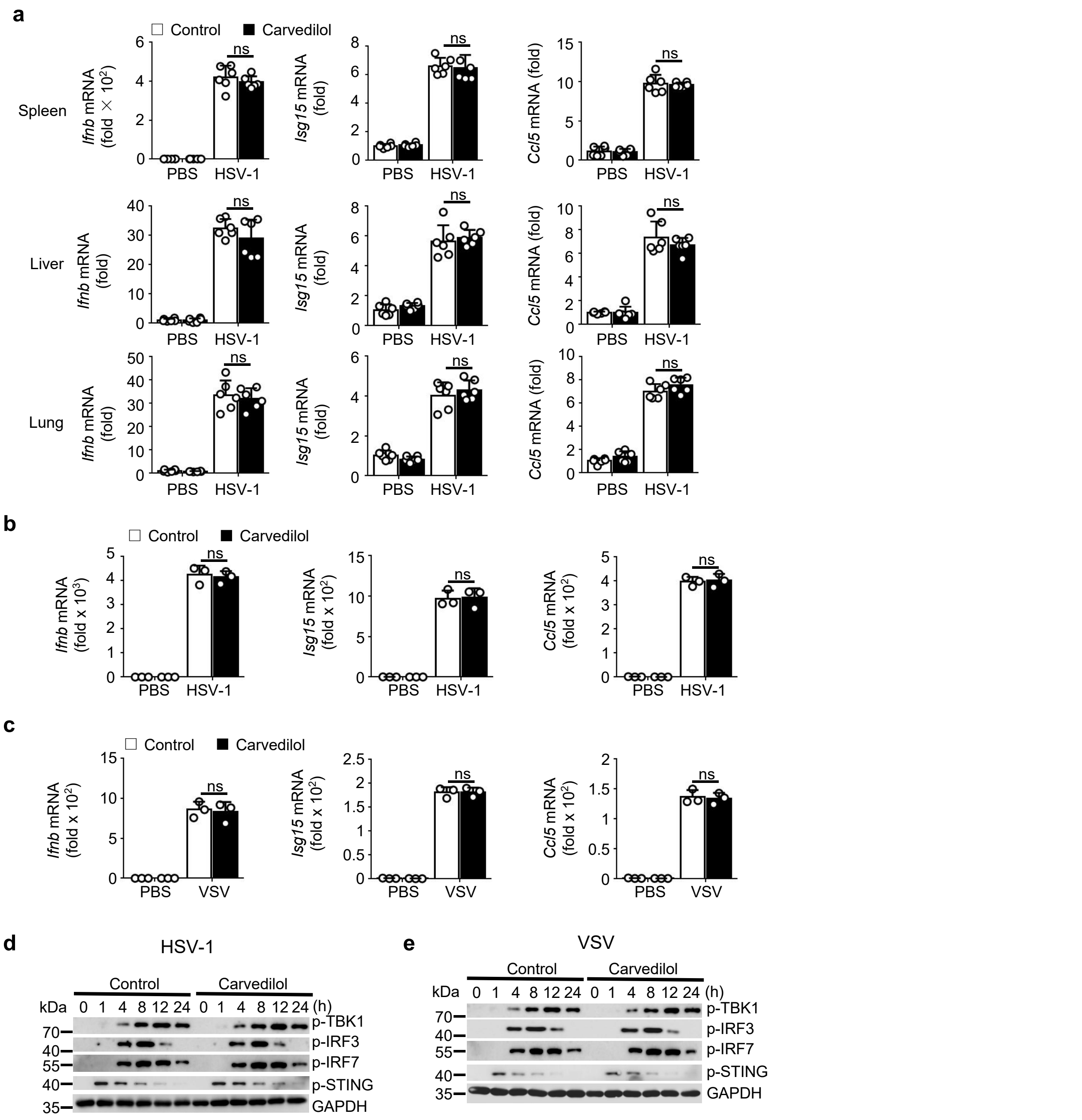

Supplementary Fig. 4: Carvedilol has no effect on *Arrb2⁻/⁻* mice and *Arrb2⁻/⁻* cells. (**a**) *Ifnb*, *Isg15*, and *Ccl5* mRNA levels in the spleens, livers, and lungs of *Arrb2⁻/⁻* 6-week-old-male mice (n = 6 per group) intraperitoneally injected with PBS or HSV-1 (2×10^7 PFU per mouse) for 24 h and then treated with PBS or carvedilol for another 24 h. (**b, c**) *fnb*, *ISG15*, and *Ccl5* mRNA in *Arrb2⁻/⁻* peritoneal macrophages infected for 16 h with HSV-1 (**b**) or VSV (**c**) and then treated with PBS or carvedilol, n = 3. (**d**, **e**) Immunoblot of lysates of peritoneal macrophages from *Arrb2⁻/⁻* mice infected with HSV-1 (**d**) or VSV (**e**) for indicated times and treated with PBS or carvedilol. Data are representative of at least three independent experiments (mean ± SEM in **a**–**c**). ns > 0.05, two-tailed unpaired Student's t-test.

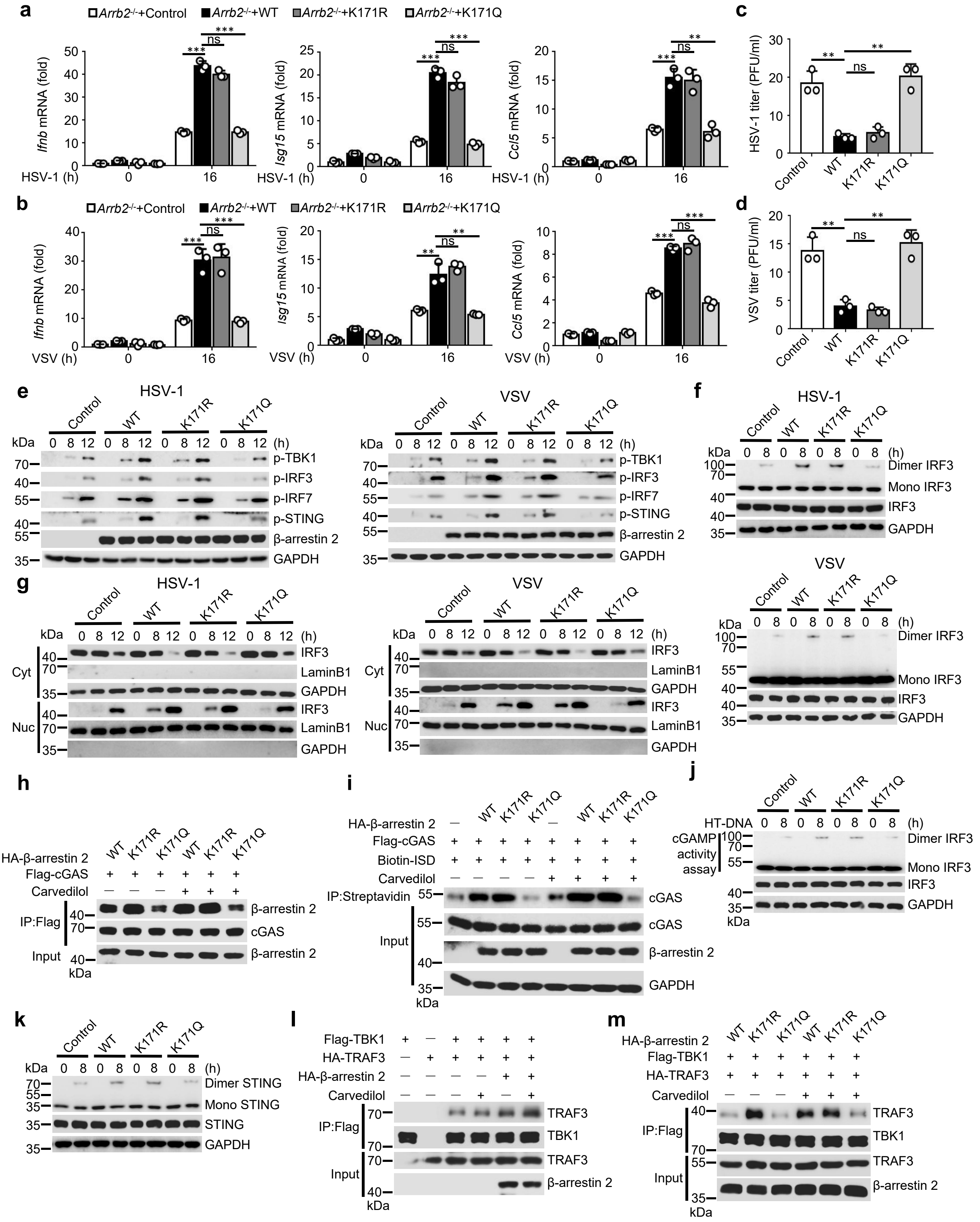

a
b
c
d
e
f
g
h
i
j
k
l
m
Arrb2-/-+Control
Arrb2-/-+WT
Arrb2-/-+K171R
Arrb2-/-+K171Q
Ifnb mRNA (fold)
Isg15 mRNA (fold)
Ccl5 mRNA (fold)
HSV-1 (h)
VSV (h)
HSV-1 titer (PFU/ml)
VSV titer (PFU/ml)
Control
WT
K171R
K171Q
HSV-1
VSV
p-TBK1
p-IRF3
p-IRF7
p-STING
β-arrestin 2
GAPDH
Dimer IRF3
Mono IRF3
IRF3
Cyt
Nuc
LaminB1
HA-β-arrestin 2
Flag-cGAS
Carvedilol
IP:Flag
Input
cGAS
Biotin-ISD
IP:Streptavidin
HT-DNA
cGAMP activity assay
Dimer STING
Mono STING
STING
Flag-TBK1
HA-TRAF3
TRAF3
TBK1
kDa
(h)

Supplementary Fig. 5: Carvedilol activated β-arrestin 2 through Lys171. (**a, b**) *Ifnb*, *Isg15*, and *Ccl5* mRNA levels in *Arrb2*$^{-/-}$ MEFs transfected with control vector, β-arrestin 2, or β-arrestin 2 mutants, infected with HSV-1 (**a**) (****P* < 0.0001, < 0.0001 in sequence, left panel; ****P* < 0.0001, < 0.0001 in sequence, middle panel; ****P* < 0.0001, ***P* = 0.0012, right panel) or VSV (**b**) (****P* = 0.0009, 0.0008 in sequence, left panel; ***P* = 0.0051, 0.0035 in sequence, middle panel; ****P* < 0.0001, < 0.0001 in sequence, right panel) for 16 h and treated with PBS or carvedilol. (**c, d**) The virus titers as in (**a**) and (**b**) for 72 h (***P* = 0.0018, 0.0011 in sequence in **c**, ***P* = 0.0031, 0.0018 in sequence in **d**). (**e**) Immunoassay of lysates of *Arrb2*$^{-/-}$ MEFs transfected as in (**a**), infected with HSV-1 (left) and VSV (right) for indicated times and treated with PBS or carvedilol. (**f**) Immunoblot analysis of monomeric and dimeric IRF3 as in (**a**) and (**b**). (**g**) Immunoblot analysis of nuclear and cytoplasmic fractions as in (**a**) and (**b**). (**h**) Immunoassay of lysates of HEK293T cells expressing various vectors. (**i**) Immunoassay of cell lysates and streptavidin-precipitated proteins from HEK293T cells transfected with various vectors and stimulated with biotin-ISD. (**j**) cGAMP activity measured by extracts from HT-DNA-stimulated (6 h) WT and *Arrb2*$^{-/-}$ peritoneal macrophages. (**k**) Immunoblot analysis of monomeric and dimeric STING in *Arrb2*$^{-/-}$ MEFs transfected as in (**a**) infected with HSV-1 for indicated times and treated with PBS or carvedilol. (**l**) Immunoassay of lysates of HEK293T cells expressing various vectors. (**m**) Immunoassay of lysates of HEK293T cells expressing various vectors. Data are representative of at least three independent experiments (mean ± SEM in **a-d,** *n* = 3). Two-tailed unpaired Student's *t*-test.

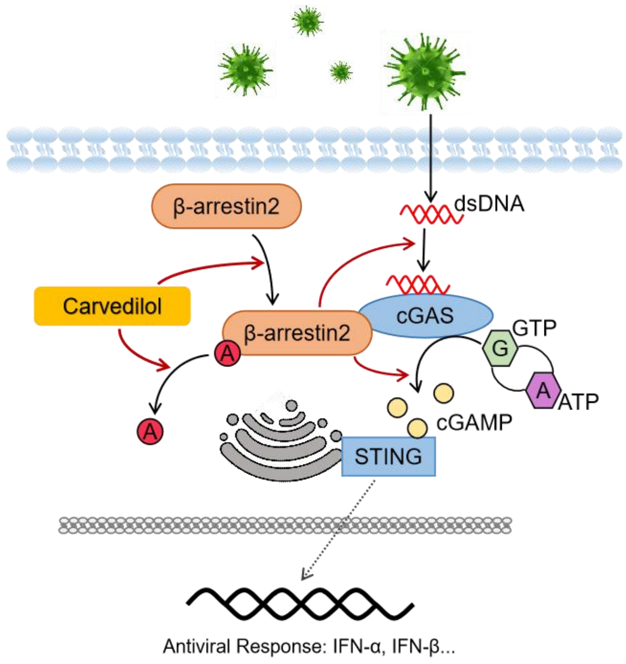

Supplementary Fig. 6:
Diagram depicting the Carvedilol–β-arrestin 2–cGAS–STING–TBK1–IRF3–Type I IFN axis.

Supplementary Table 1: Primer sequences used to amplify human gene and mouse gene in real-time quantitative PCR.

Human *Ifnb*	forward	TCTGGCACAACAGGTAGTAGGC
	reverse	GAGAAGCACAACAGGAG
Human *Cxcl10*	forward	GGAACCTCCAGTCTCAGCACCA
	reverse	AGACATCTCTTCTCACCCTTC
Human *Isg15*	forward	TTTGCCAGTACAGGAGCTTGTG
	reverse	GGGTGATCTGCGCCTTCA
Human *GAPDH*	forward	GCAAATTCCATGGCACCGT
	reverse	GCCCCACTTGATTTTGGAGG
mouse *Ifnb*	forward	AGTTACACTGCCTTTGCC
	reverse	GTTGAGGACATCTCCCAC
mouse *Cxcl10*	forward	CCAAGTGCTGCCGTCATTTT
	reverse	GATAGGCTCGCAGGGATGAT
mouse *Isg15*	forward	GGTGTCCGTGACTAACTCCAT
	reverse	TGGAAAGGGTAAGACCGTCCT
mouse *Gapdh*	forward	CCCACTAACATCAAATGGGG
	reverse	CCTTCCACAATGCCAAAGTT
